# Clinical and experimental studies regarding the expression and diagnostic value of carcinoembryonic antigen-related cell adhesion molecule 1 in non-small-cell lung cancer

**DOI:** 10.1186/1471-2407-13-359

**Published:** 2013-07-25

**Authors:** Mu-qing Zhou, Yan Du, Yi-wen Liu, Ying-zhi Wang, Yi-qing He, Cui-xia Yang, Wen-juan Wang, Feng Gao

**Affiliations:** 1 Department of Clinical Laboratory, the Sixth People’s Hospital, Shanghai Jiao-tong University School of Medicine, 600 Yi-shan Road, Shanghai 200233, People's Republic of China; 2 Department of Molecular Biology Laboratory, the Sixth People’s Hospital, Shanghai Jiao-tong University School of Medicine, 600 Yi-shan Road, Shanghai 200233, People's Republic of China

**Keywords:** Carcinoembryonic antigen-related cell adhesion molecule 1, Non-small-cell lung carcinomas, Enzyme-linked immunosorbent assay, Receiver operating characteristic curve

## Abstract

**Background:**

Carcinoembryonic antigen-related cell adhesion molecule 1 (CEACAM1) is a multifunctional Ig-like cell adhesion molecule that has a wide range of biological functions. According to previous reports, serum CEACAM1 is dysregulated in different malignant tumours and associated with tumour progression. However, the serum CEACAM1 expression in non-small-cell lung carcinomas (NSCLC) is unclear. The different expression ratio of CEACAM1-S and CEACAM1-L isoform has seldom been investigated in NSCLC. This research is intended to study the serum CEACAM1 and the ratio of CEACAM1-S/L isoforms in NSCLC.

**Methods:**

The expression of the serum CEACAM1 was determined by enzyme-linked immunosorbent assay. The protein expression and the location of CEACAM1 in tumours were observed by immunohistochemical staining. The CEACAM1 mRNA levels in tumour and normal adjacent tissues were measured using quantitative real-time PCR, and the expression patterns and the rate of CEACAM1-S and CEACAM1-L were analysed by reverse transcription-PCR.

**Results:**

Serum CEACAM1 levels were significantly higher in NSCLC patients compared with that from normal healthy controls (*P* <0.0001). 17 patients (81%) among 21 showed high expression of CEACAM1 by immunohistochemical staining. Although no significant differences were found between tumour and normal tissues on mRNA expression levels of CEACAM1 (*P* >0.05), the CEACAM1-S and the CEACAM1-S/L (S: L) ratios were significantly higher in tumour than normal tissues (*P* <0.05).

**Conclusions:**

Our data indicated that the serum levels of CEACAM1 could discriminate lung cancer patients from health donors and that CEACAM1 might be a useful marker in early diagnosis of NSCLC. Moreover, our results showed that the expression patterns of CEACAM1 isoforms could be changed during oncogenesis, even when total CEACAM1 in tumour tissues did not show significant changes. Our study suggested that the expression ratios of CEACAM1-S/CEACAM1-L might be a better diagnostic indicator in NSCLC than the quantitative changes of CEACAM1.

## Background

Lung cancer is currently the most common cancer in terms of incidence and mortality worldwide [1]. Non-small-cell lung carcinoma (NSCLC) accounts for over 80% of all histological lung cancers. Approximately 40% of patients with NSCLC show locally advanced disease with lymph node involvement at the time of diagnosis. Thus, early NSCLC detection is highly valuable.

Carcinoembryonic antigen-related cell adhesion molecule 1 (CEACAM1), a single-pass transmembrane type I glycoprotein, belongs to the carcinoembryonic antigen (CEA) family. This protein is widely expressed in a variety of proliferating and quiescent epithelial, endothelial, and haematopoietic cells [2]. CEACAM1 is involved in a variety of cell biological events, such as morphogenesis [3], vasculogenesis [4], cell motility [5], cell proliferation [6,7], infection, and inflammation [2]. CEACAM1 exists in 11 known isoforms, resulting from differential splicing and proteolytic processing. The functions of the 11 known CEACAM1 isoforms are divided based on the isoforms CEACAM1-L and CEACAM1-S, which are named based on the length of their cytoplasmic tail. The L-form contains two immunoreceptor tyrosine-based inhibitory motifs (ITIMs), whereas the S-form does not. Both isoforms are co-expressed in most CEACAM1-expressing tissues, and the ratio between the two isoforms determines the signalling outcome [8-12].

Aberrant CEACAM1 expression is associated with tumour progression and has been found in a variety of human malignancies. Previous reports showed that CEACAM1 is down-regulated in many types of tumours, such as colorectal carcinoma [13], hepatoma [14], breast carcinoma [15], renal cell carcinoma [16] and prostate carcinoma [17]. Additionally, the inhibition of tumour growth upon CEACAM1 re-expression in tumour cells was reported to led to the original definition of CEACAM1 as a tumour suppressor [18]. In contrast, CEACAM1 was also found to be up-regulated in malignant melanoma [19], thyroid cancer [20] and gastric adenocarcinoma. Although the published literature on CEACAM1 expression in cancer is contradictory, most investigators agree that these changes in expression offer an important indicator for clinical diagnoses.

Recent reports have shown that the serum CEACAM1 level was increased in pancreatic adenocarcinoma [21] and melanoma patients [19,22]. This increase was correlated with disease progression, providing evidence for the potential value of soluble CEACAM1 as a tumour marker. In lung cancer, mainly immunohistochemical evidence has accumulated indicating that epithelial CEACAM1 expression is associated with tumour metastasis and progression [23-26]. However, little information exists concerning serum CEACAM1 in lung cancer. This information would be valuable because currently available circulating tumour markers for lung cancer, such as carcinoembryonic antigen (CEA) and neuron-specific enolase (NSE), are not satisfactory, and the need for better prognostic markers is urgent. It is therefore necessary to evaluate the changes in serum CEACAM1 in the context of lung cancer.

In an exploratory phase (phase I) study [27] in which the serum level of CEACAM1 was evaluated to determine whether it could be used to discriminate lung cancer patients from health donors, we set our sample size as recommended by Obuchowski et al. [28]. We compared the serum level of CEACAM1 in NSCLC patients with healthy donors and analyzed the location and expression of CEACAM1 in primary tumour tissues by immunohistochemical staining. Additionally, the CEACAM1 expression levels in lung cancer tissues together with adjacent normal lung tissues were verified at the mRNA level from the same serum providers in parallel using quantitative real-time PCR. The CEACAM1 S/L isoform expression patterns were verified by reverse transcription-PCR.

## Methods

### Serum and tissue samples

A total of 69 serum samples were included in this study, including 35 samples that were collected from NSCLC patients before surgery (average age: 60, range: 34 to 78 years; samples with any other disease outside the lungs were excluded) and 34 samples that were collected from sex- and age-matched healthy volunteers who passed all of the routine medical examinations (e.g., blood tests, X-ray, and computerised tomography test) without abnormal results. Informed consent was obtained from all of the patients who participated in the study, which was conducted with the approval of the Ethics Committee of the Scientific and Ethical Committee of Shanghai Jiao Tong University in accordance with the Helsinki declaration of 1975 (as revised in 1983). All the patients were diagnosed with cancer for the first time, and none previously received chemotherapy or radiation therapy.

Briefly, the samples were maintained at room temperature for approximately 30 minutes and then centrifuged at 1,300 g at 4°C for 20 minutes. The serum was then collected, divided into aliquots and frozen at -80°C until analysis. We also collected 21 paired (tumour and normal mucosa) tissue samples in parallel from the same 35 NSCLC patients mentioned above to assay by quantitative real-time polymerase chain reaction (qRT-PCR). During the surgical removal of each tumour, an adjacent section of normal mucosa was also removed for normal background tissue following pathological confirmation that it was free from tumour deposits. Tissues were obtained and snap frozen in liquid nitrogen. Additionally, 13 of 21 pairs of tissues were analysed for CEACAM1 isoforms: 6 cases of adenocarcinoma, 5 cases of squamous cell carcinoma, 1 case of poorly differentiated carcinoma, and 1 case of a lymphoepithelioma-like carcinoma. Detailed clinical and pathological information of the samples can be found in Additional file 1: Table S1.

### Sandwich ELISA for serum CEACAM1

Serum CEACAM1 was analysed with enzyme-linked immunosorbent assay (ELISA) kits (RayBiotech, Atlanta, GA, USA) according to the manufacturer's instructions. Briefly, a 96-well microplate was precoated with anti-human CEACAM1, which recognises the extracellular domain (a.a. 35-428). Before use, all of the reagents and samples were brought to room temperature (18-25°C). The standard dilution series ranged from 20.58 to 15,000 pg/ml. First, 100 μl of each standard or serum sample (1:100 prediluted) was added to the appropriate wells and incubated for 2.5 hours at 24°C with gentle shaking. After discarding the solution and washing 4 times, 100 μl of prepared biotinylated anti-human CEACAM1 antibody was added to each well and incubated for 1 hour. After washing away unbound biotinylated antibody, 100 μl of horseradish peroxidase (HRP)-conjugated streptavidin was pipetted into the wells and incubated for 45 minutes, and 100 μl of 3,3’ ,5,5’-Tetramethylbenzidine (TMB) one-step substrate reagent was added after 5 washes. Subsequently, 50 μl of stop solution was added to each well, and the plate was immediately read at 450 nm.

In addition, we assayed carcinoembryonic antigen (CEA; ARCHITECT i2000 SR, Abbott, Chicago, IL, USA) and neuron-specific enolase (NSE; cobas e601 Roche, Basel, N.A., Switzerland) in all of the serum samples. The CEA and NSE cut-off values were 5.0 and 17 ng/ml, respectively. Tumour markers with serum values higher than the cut-off were classified as positive, while those with values lower than the cut-off were negative.

### Immunohistochemical staining

To determine the expression and location of CEACAM1, a total of 21 specimens were stained using immunohistochemistry method, which was performed on paraffin-embedded specimens with the mouse monoclonal anti-CEACAM1 antibody (Abcam; 29H2, Cambridge, UK, dilution: 1:75). Briefly, the sections were dewaxed, and endogenous peroxidase was blocked by immersing the slides in a 3% solution of hydrogen peroxide in methanol for 10 minutes followed by antigen retrieval. The slides were microwaved for 10 minutes in 10 mmol/L citrate buffer, pH 6.0. After washing 3 times in 1 mol/l phosphate-buffered saline (PBS; pH 7.4) for 5 minutes, the sections were blocked with normal rabbit serum in a humidity chamber for 30 minutes at room temperature. The excess serum was rinsed off with 1 mol/l PBS, and the sections were incubated with primary antibodies in a humidity chamber overnight at 4°C. The next morning, sections were rinsed with PBS before incubation with the biotinylated second antibody in a humidity chamber for 40 minutes at 37°C. After rinsing with PBS, the streptavidin–peroxidase complex reagent (StrepABComplex/HRP Duet, DAKO, Glostrup, Denmark) was added. Diaminobenzidine and hydrogen peroxide were used for visualisation. For negative controls, the primary antibody was replaced with PBS.

**Figure 1:**
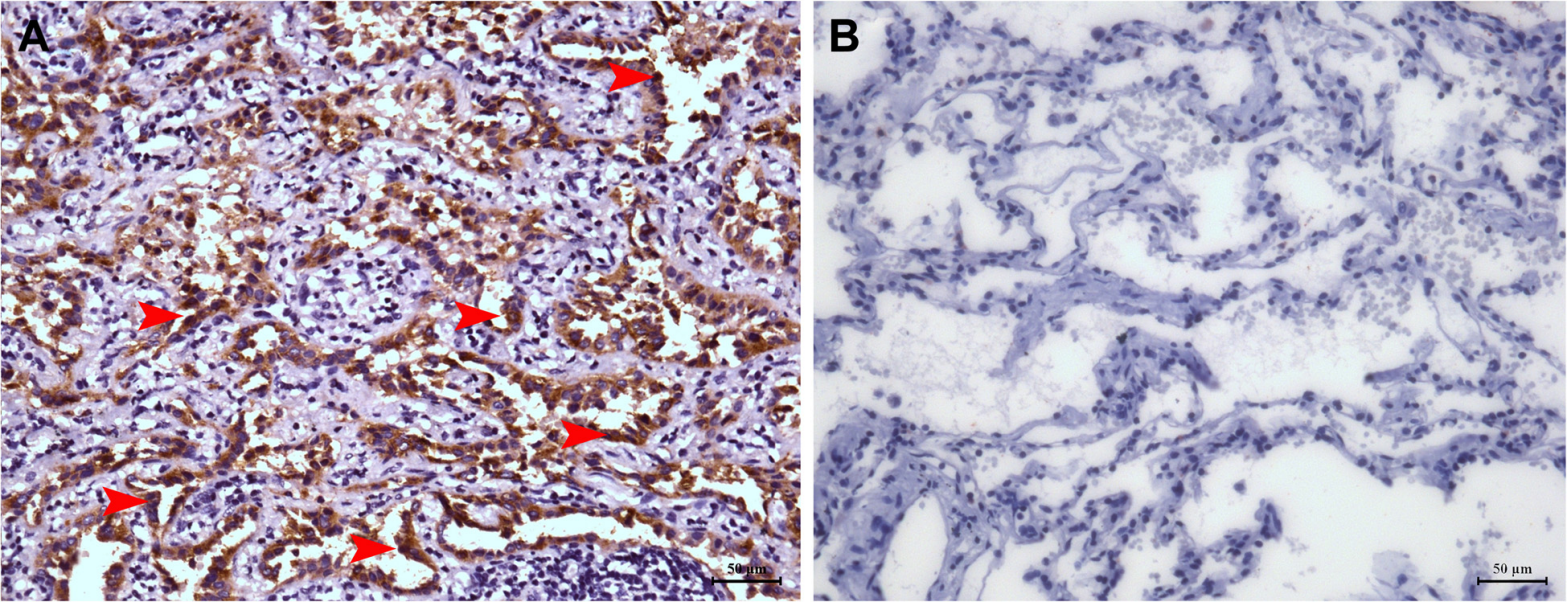
**Immunohistochemical staining of CEACAM1 in primary NSCLC. (A)** Representative CEACAM1 staining in tumour tissues. Arrows indicate the positive staining of neoplastic epithelium cells (brown colour, 200 × microscopic field). **(B)** No specific staining is visible in the section of normal cells adjacent to the tumour samples (200 × microscopic field).

All the 21 NSCLC were categorized into a high expression group (i.e., ≥ 66% positive tumour cells) and a low expression group (i.e., < 66% positive tumour cells) according to the percentage of positive tumour cells [23,29,30] (Figure 1). The high or low classifications were independently assigned by two experienced pathologists and consensus was achieved after discussion.

### Quantitative real-time polymerase chain reaction for the CEACAM1 RNA level

Total RNA was isolated using the TRIzol reagent (Invitrogen, Carlsbad, CA, USA) according to the manufacturer’s instructions. The purity and concentration of RNA were determined with a spectrophotometer at 260 nm. Complementary DNA (cDNA) was generated by the PrimeScript® RT reagent Kit (Takara Biotechnology, Otsu, Shiga, Japan). GAPDH was used as an internal control for each sample. Quantitative real-time PCR was performed using specific primers (Table 1). Briefly, 1 μg of total RNA was denatured for 5 minutes at 70°C and cooled for 5 minutes on ice. Reverse transcriptase (RT) was added to a total volume of 20 μl, and reverse transcription was performed for 15 minutes at 37°C followed by 5 seconds at 85°C, according to the protocol recommended by the manufacturer. The synthesised cDNA was either used immediately for PCR amplification or stored at -20°C for further analysis. Quantitative PCR was performed in a total reaction volume of 20 μl per capillary in the LightCycler format for 40 cycles (denaturation: 30 seconds at 95°C, annealing: 20 seconds at 60°C, and extension: 15 seconds at 72°C). After PCR amplification, melting curve analysis was performed to verify the specificity of the test.

**Table 1:** Primer sequences for real-time PCR and reverse transcription-PCR

**Primer name**		**Primer sequences**	**Product**
**Real-time PCR**	CEACAM1^*^	**F:5’-CAGTCACCTTGAATGTCACCTATG-3’**	**144 bp**
		**R:5’-GTTCCATTGATAAGCCAGGAGTAC-3**	
	**GAPDH**	**F: 5’-GCACCGTCAAGGCTGAGAAC-3’**	**142 bp**
	*NM_002046.*	**R: 5’-ATGGTGGTGAAGACGCCAGT-3’**	
**Reverse transcription-PCR**	CEACAM1^*^	**F:5’-GGTTGCTCTGATAGCAGTAG-3’**	**408 bp (L-form)**
		**R: 5’-AGCCTGGAGATGCCTATTAG-3’**	**355 bp (S-form)**
	**GAPDH**	**F:5’-GGGAAGGTGAAGGTCGGAGTC-3’**	**570 bp**
	*NM_002046.*	**R:5’-AGGGGCCATCCACAGTCTTCT-3’**	

“*” The GenBank accession numbers *NM_001712.4, NM_001184815.1*, *NM_001184815.1, NM_001184813.1, NM_001184816.1* and *NM_001205344.1* correspond to CEACAM1 transcript variants 1, 2, 3, 4, 5, 6, respectively.

### Reverse transcription-PCR for the expression patterns of CEACAM1 isoforms

The reverse transcription PCR primers as reported by Wang et al. [31] and Gaur et al. [32] (Table 1) were designed to distinguish the 408 bp (CEACAM1-L) and 355 bp (CEACAM1-S) CEACAM1 isoforms. The PCR was performed in a total volume of 50 μl containing 0.25 μl of TaKaRa Ex Taq (5 U/μl), 5 μl of 10× Ex Taq Buffer (Mg^2+^ Plus), 4 μl of dNTP mixture, 2 μl of cDNA template, 1 μl of each forward and reverse primer and ddH_2_O. After pre-denaturation at 94°C for 5 minutes, the PCR cycling conditions for CEACAM1 and GAPDH were as follows: 30 cycles of 94°C for 1 minute, 60°C for 30 seconds, and 72°C for 30 seconds. The reaction was performed with an Eppendorf thermal cycler (Eppendorf, Hamburg, Germany). At the end of the reaction, the mixtures were loaded onto a 2% agarose gel and stained with ethidium bromide prior to examination under UV light.

### Statistical analysis

L-form CEACAM1 and S-form CEACAM1 levels were represented as integral optical density (IOD) values with Image Pro Plus V6.0 for Windows (Media Cybernetics, Inc., Rockville, MD, USA). Briefly, after intensity rectification, IODs were obtained as the ratio of sum optical density (OD) to the sum area, which is proportional to the quantity of RNA. Most of the data were not normally distributed. Thus, they were expressed as a median or a range. The Mann–Whitney and Kruskal–Wallis tests were used to determine the significance of two independent groups and various groups, respectively. Nonparametric receiver operating characteristic (ROC) curves in which the value for sensitivity is plotted against the false-positive rate (1-specificity) were generated to assess the diagnostic accuracy of serum CEACAM1. Receiver operating characteristic (ROC) curves are measured to test whether the area under the curve (AUC) of the ROC exceeds 0.5. If not, no further assessment of the diagnostic test is warranted. Statistical significance in this study was set at *P* < 0.05, and all of the reported *P* values are 2-sided. All of the analyses were performed with SPSS v.16 for Windows (SPSS Inc., Chicago, IL, USA) or SigmaPlot V. 12 for Windows (Systat Software Inc., San Jose, CA, USA).

## Results

### CEACAM1 serum levels

**Figure 2:**
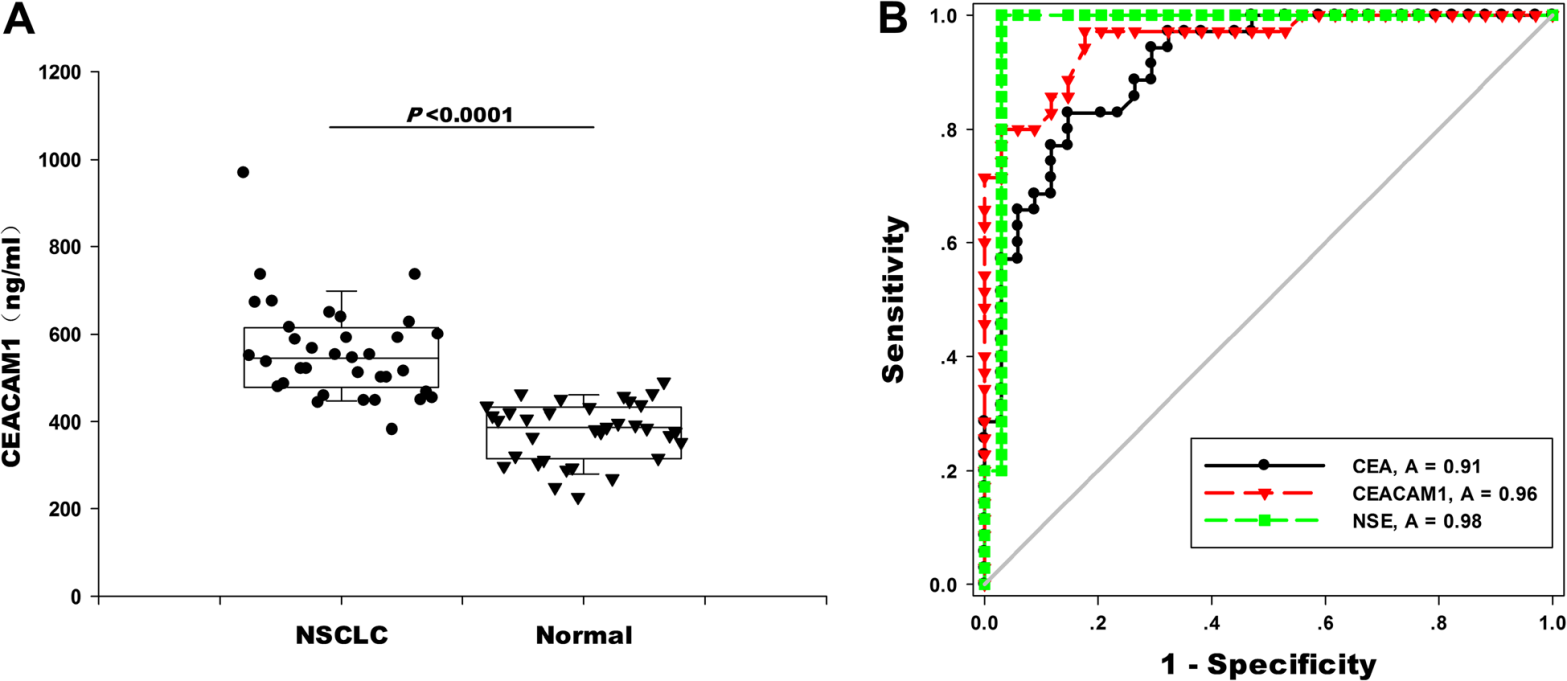
**Individual CEACAM1 serum levels and receiver operator characteristic curves for NSCLC patients and normal controls. (A)** The serum CEACAM1 in patients with NSCLC and normal controls are plotted as a distribution (*P* < 0.001). **(B)** ROC curves generated from the serum CEACAM1, CEA and NSE of 35 patients with NSCLC. The areas under the curves are 0.96, 0.91 and 0.98 for CEACAM1, CEA and NSE, respectively (*P* < 0.05).

The clinical and pathological characteristics of patients are shown in Table 2. The median serum CEACAM1 level was significantly higher in patients with NSCLC compared with normal healthy controls (*P* < 0.0001; Figure 2A). For patients with NSCLC, the median CEACAM1 level was 544.79 ng/ml (range: 381.30 ~ 968.13 ng/ml), and for normal controls, the median was 386.20 ng/ml (range: 226.80 ~ 490.11 ng/ml). Patients who were at an early stage of disease (stage I and II disease) showed significantly higher CEACAM1 levels than patients in stage III and IV (*P* = 0.016; Table 2). Moreover, serum CEACAM1 levels were significantly lower in female patients than in male patients (Table 2).

**Table 2:** Association between the CEACAM1 serum expression levels and clinical parameters

**Groups**	**No.**	**CEACAM1**		
		**Median (P**_**50**_**)**	**Range**	***P *value**
**Age**				
**<60**	**16**	**550.70**	**381.30 ~ 735.84**	**0.679**
**≥60**	**19**	**535.32**	**442.07 ~ 968.13**	
**Sex**				
**Male**	**16**	**588.98**	**442.07 ~ 968.13**	0.042*
**Female**	**19**	**513.95**	**381.30 ~ 735.84**	
**Location**				
**Left**	**11**	**519.89**	**381.30 ~ 674.69**	**0.619**
**Right**	**24**	**548.34**	**442.07 ~ 968.13**	
Staging**				
**Stage Ia ~ IIb**	**19**	**566.04**	**381.03 ~ 968.13**	0.016*
**Stage IIIa ~ IV**	**16**	**499.66**	**442.07 ~ 638.68**	
**Grading**				
**G1 ~ 2**	**14**	**542.42**	**442.07 ~ 968.13**	**0.775**
**G3 ~ 4**	**21**	**544.79**	**381.30 ~ 735.84**	
**Histology**				
**Squamous cell carcinoma**	**6**	**571.31**	**499.66 ~ 627.02**	**0.180**
**Adenocarcinoma**	**19**	**519.89**	**381.30 ~ 735.84**	
Others***	**10**	**579.29**	**442.07 ~ 968.13**	
**Lymph node metastasis**				
**Node negative**	**13**	**566.04**	**381.30 ~ 968.13**	**0.274**
**Node positive**	**22**	**532.34**	**442.07 ~ 735.84**	
**Invasion depth**				
**pT1 ~ pT2**	**26**	**551.88**	**381.30 ~ 968.13**	**0.131**
**pT3 ~ pT4**	**9**	**519.89**	**442.07 ~ 638.68**	

*P < 0.05.

“**” 7^th^ edition of the TNM classification of lung cancer by the International Association for the Study of Lung Cancer (IASLC).

“***” Others represent 5 poorly differentiated carcinomas, 2 mixed histologies, 2 neuroendocrine carcinomas and 1 lymphoepithelioma-like carcinoma.

In multivariable logistic regression analysis, CEACAM1 levels significantly predicted NSCLC vs. normal control (OR: 1.052; 95% CI: 1.022 ~ 1.083; *P* < 0.001) when adjusted for age and gender effects. The ability of serum CEACAM1, CEA and NSE to predict NSCLC was analysed by nonparametric ROC analyses. When used to distinguish NSCLC from normal healthy individuals, the AUCs for serum CEACAM1, CEA and NSE were 0.96 (95% CI: 0.9148 ~ 0.9995; *P* < 0.001), 0.91 (95% CI: 0.8454 ~ 0.9773; *P* < 0.001) and 0.98 (95% CI: 0.9302 ~ 1.023; *P* < 0.001), respectively. Using the cut-off level of 440.3 ng/ml (according to the Youden index), serum CEACAM1 had a sensitivity of 97%, a specificity of 82%, a positive predictive value of 70%, and a negative predictive value of 95% (Figure 2B, Additional file 2: Table S2). Therefore, according to the AUC of the ROC curve, CEACAM1 was better than CEA but did not exceed NSE.

In the clinic, the cut off values for CEA and NSE were set to 5 and 17 ng/ml, respectively. The corresponding diagnostic accuracy of CEACAM1, CEA and NSE is summarised in Additional file 2: Table S2. Generally, CEACAM1 had a significantly higher sensitivity (97%) than CEA (29%, *P* < 0.05) and NSE (20%, *P* < 0.05). However, CEA (97%) and NSE (97%) had better specificity than CEACAM1 (82%), not significantly (*P* > 0.05). The negative predictive value of CEACAM1 (95%) was higher compared with CEA (57%) and NSE (54%), but the positive predictive value of CEACAM1 (70%) was lower than CEA (91%) and NSE (88%), which is likely a result of the low sensitivity of CEA and NSE.

### CEACAM1 protein and mRNA expression in lung tissues

By immunohistochemical staining, we found that the CEACAM1 expression was restricted to neoplastic epithelium in all the specimens of 21 patients (Figure 1A). No CEACAM1 expression was found in normal cells adjacent to the tumours or in the negative controls (Figure 1B). Tumours of 17 patients (81%) were classified as high expression (Figure 1A, i.e., ≥66% positive tumour cells), and specimens of 4 patients (19%) were classified as low expression (Figure 1B, i.e., <66% positive tumour cells).

**Figure 3:**
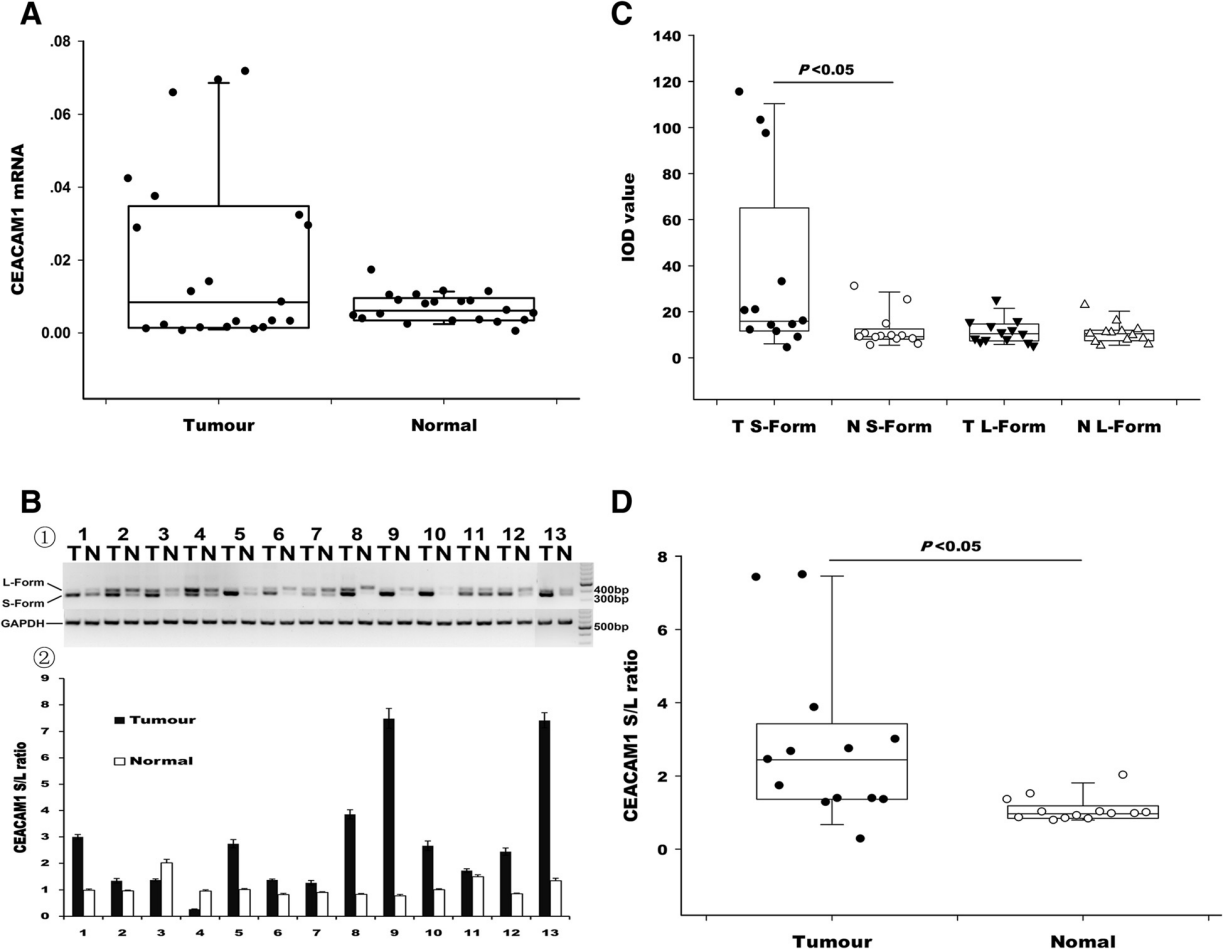
**The S-form and L-form CEACAM1 mRNA expression level and patterns in NSCLC and normal tissues. (A)** The expression level of CEACAM1 mRNA in tumour tissues and normal tissues (*P* > 0.05). **(B)** ①. Representative RT-PCR data performed with total RNA extracts from 13 paired NSCLC specimens (T = tumour, N = normal). ②. Histogram depicting CEACAM1-S/CEACAM1-L (S: L) ratio for the 13 paired specimens in bar graph quantified with Image Pro Plus program. Data represent the mean ± SD of at least three independent experiments. **(C)** The distribution of integral optical density (IOD) values for CEACAM1-S form and L-form compared with GAPDH in 13 paired tumour and normal adjacent tissues samples (T = tumour; N = normal). **(D)** The distribution of the CEACAM1-S/L ratio in 13 paired tumour and normal adjacent tissues samples (*P* < 0.05).

The 2^-△Ct^ (-△∆Ct = Ct,_ GAPDH_-Ct,_ CEACAM1_) method was employed. Although 15 of 21 subjects showed higher CEACAM1 mRNA levels in tumours compared to adjacent tumour-free tissues, no significant differences were found between the mRNA expression of CEACAM1 in tumours and normal tissues by the Wilcoxon signed-rank test for 2 related samples (Figure 3A). Further studies of CEACAM1 mRNA levels and the patient clinical and pathological characteristics were shown in Table 3. CEACAM1 mRNA levels were significantly higher in male patients than in female patients (*P* = 0.028), which was consistent with the serum protein levels (Table 2). CEACAM1 mRNA levels also showed a significant negative correlation with tumour invasive extension (*P* = 0.039), which is in accordance with the serum protein levels. In addition, patients with adenocarcinoma showed higher CEACAM1 mRNA levels than squamous cell carcinoma or other types (*P* = 0.003). No other significant association was found between clinical characteristics and CEACAM1 mRNA levels.

**Table 3:** Association between the CEACAM1 mRNA expression patterns and clinical parameters

**Groups**	**No.**	**Tumour tissue**			**Normal tissue**		
		**Median (P**_**50**_**)**	**Range**	***P *value**	**Median (P**_**50**_**)**	**Range**	***P *value**
**Age**							
**≥60**	**13**	**0.0293**	**5.60 × 10**^**-4**^ **~ 0.0716**	**0.082**	**5.31 × 10**^**-3**^	**2.28 × 10**^**-3**^ **~ 0.0172**	**0.828**
**<60**	**8**	**2.62 × 10**^**-3**^	**9.25 × 10**^**-4**^ **~ 0.0287**		**6.99 × 10**^**-3**^	**3.80 × 10**^**-4**^ **~ 0.0113**	
**Sex**							
**Male**	**12**	**3.09 × 10**^**-3**^	**5.60 × 10**^**-4**^ **~ 0.0373**	0.028*	**4.31 × 10**^**-3**^	**3.80 × 10**^**-4**^ **~ 0.0172**	**0.088**
**Female**	**9**	**0.0293**	**1.36 × 10**^**-3**^ **~ 0.0716**		**8.67 × 10**^**-3**^	**3.81 × 10**^**-3**^ **~ 0.0114**	
Staging***							
**Stage Ia ~ IIb**	**14**	**0.0126**	**5.60 × 10**^**-4**^ **~ 0.0693**	**0.332**	**5.61 × 10**^**-3**^	**5.60 × 10**^**-4**^ **~ 0.0693**	**0.709**
**Stage IIIa ~ IV**	**7**	**2.09 × 10**^**-3**^	**9.24 × 10**^**-4**^ **~ 0.716**		**7.87 × 10**^**-3**^	**2.85 × 10**^**-3**^ **~ 0.0113**	
**Grading**							
**G1 ~ 2**	**10**	**0.0126**	**1.04 × 10**^**-3**^ **~ 0.716**	**0.439**	**8.60 × 10**^**-3**^	**3.20 × 10**^**-3**^ **~ 0.172**	0.020*
**G3 ~ 4**	**11**	**3.21 × 10**^**-3**^	**5.60 × 10**^**-4**^ **~ 0.422**		**3.81 × 10**^**-3**^	**3.80 × 10**^**-4**^ **~ 0.0113**	
**Histology**							
**Squamous**^**1**^	**5**	**0.00144**	**9.246 × 10**^**-4**^ **~ 0.0032**		**0.0032**	**3.80 × 10**^**-4**^ **~ 0.0171**	
**Adenocarcinoma**	**11**	**0.0286**	**0.0013 ~ 0.072**	0.003*	**0.00787**	**0.0034 ~ 0.0114**	**0.585**
Others**	**5**	**0.0021**	**5.60 × 10**^**-4**^ **~ 0.014**		**0.00853**	**0.0023 ~ 0.011**	
**Lymph node metastasis**							
**node negative**	**7**	**0.0140**	**1.04 × 10**^**-3**^ **~ 0.0658**	**0.502**	**8.53 × 10**^**-3**^	**3.81 × 10**^**-3**^ **~ 0.017**	**0.117**
**node positive**	**14**	**3.19 × 10**^**-3**^	**5.60 × 10**^**-4**^ **~ 0.0716**		**4.98 × 10**^**-3**^	**3.80 × 10**^**-4**^ **~ 0.011**	
**Invasion depth**							
**pT1 ~ pT2**	**17**	**0.0129**	**5.60 × 10**^**-4**^ **~ 0.0716**	0.039*	**2.12 × 10**^**-3**^	**3.80 × 10**^**-4**^ **~ 0.0172**	**0.720**
**pT3 ~ pT4**	**4**	**1.63 × 10**^**-3**^	**9.25 × 10**^**-4**^ **~ 3.21 × 10**^**-3**^		**6.28 × 10**^**-3**^	**2.85 × 10**^**-3**^ **~ 0.0113**	

“1” represents squamous cell carcinoma; *P < 0.05.

“**” Others represents 2 poorly differentiated carcinomas, 1 mixed histology, 1 neuroendocrine carcinoma and 1 lymphoepithelioma-like carcinoma.

“***” 7^th^ edition of the TNM classification of lung cancer by the International Association for the Study of Lung Cancer (IASLC).

### CEACAM1 isoform expression patterns

To determine whether the expression of CEACAM1-L and CEACAM1-S are altered in primary NSCLC, we analysed 13 pairs of primary tumour and normal lung tissue specimens with the same PCR primers reported by Gaur et al. [32] and Wang et al. [31]. The forward primer is located in exon 6, and the reverse primer is located within the 3’ untranslated region. Thus, the primers can amplify a 408 bp fragment (CEACAM1-L) or a 355 bp fragment (CEACAM1-S) simultaneously by inclusion or exclusion of exon 7. As shown in Figure 3B, CEACAM1-S was predominantly expressed in lung tumours tissues, whereas the normal tissues predominantly expressed CEACAM1-L. In total, 12 of 13 lung tumours had a CEACAM1-S/CEACAM1-L (S: L) ratio greater than 1 (S-form > L-form), whereas normal lung tissue did not. Further statistical data were consistent with this finding. The CEACAM1-S and the CEACAM1-S/CEACAM1-L (S: L) ratio was significantly higher in tumours than in normal tissues (*P* = 0.023 for CEACAM1-S and 0.016 for the CEACAM1-S/CEACAM1-L (S: L) ratio; Figure 3C and 3D, Table 4). However, the expression of CEACAM1-L did not show a marked difference between tumour and normal tissue (Figure 3C, Table 4).

**Table 4:** Expression patterns for the CEACAM1 S and L forms in NSCLC tissues

**Groups**	**Median**	**95% CI**	***P *value**
Tumour S-form (IOD)	15.89	11.95 ~ 60.44	0.023*
Normal S-form (IOD)	9.16	7.23 ~ 16.45	
Tumour L-form (IOD)	10.43	8.05 ~ 14.58	0.917
Normal L-form (IOD)	10.45	7.92 ~ 13.60	
Tumour (S:L) ratio	2.44	1.49 ~ 4.20	0.016*
Normal (S:L) ratio	0.96	0.86 ~ 1.28	

*P < 0.05.

## Discussion

CEACAM1 mediates various key signal transduction pathways in tumour progression [8,33-35]. Reports indicated that increased CEACAM1 is strongly associated with NSCLC and correlated with metastasis and progression, and its expression could be determined in tumour tissue by immunohistochemistry. However, as an invasive examination, tissue biopsy has obvious limitations in the application of CEACAM1 as an early diagnosis marker in the clinic. Recently, soluble CEACAM1 has been found in body fluids, including saliva, serum, seminal fluid, and bile [36,37]. However, there are few reports concerning the diagnostic value of circulating CEACAM1 in lung cancer patients, although the early diagnosis of NSCLC is unsatisfactory.

In this study, we aimed to study whether CEACAM1 could discriminate lung cancer patients from health donors [27]. ROC analysis showed that the AUC for CEACAM1 remarkably exceeded 0.5 at 0.96, which strongly suggests the promising future of CEACAM1 as a tumour monitor. Furthermore, 17 of 21 (81%) patients showed high expression for CEACAM1 in lung tumours, and CEACAM1 expression was restricted to neoplastic epithelium, indicating that CEACAM1 was associated with NSCLC. Although CEACAM1 mRNA levels did not show a statistically significant difference between tumour and normal lung tissues, the expression of CEACAM1-S and the S/L ratios in tumour tissues showed remarkable changes during oncogenesis.

Our results suggest that CEACAM1 is associated with an increased risk for NSCLC and could reflect disease burden (Figure 2A and Table 2). Based on the data of AUC, the serum level of CEACAM1 ranked between that of CEA and NSE and could provide complementary evidence to these markers. With regards to distinguishing between individuals with a cut off value, CEACAM1 demonstrated hypersensitivity and a negative predictive value (Additional file 2: Table S2), indicating that CEACAM1 may outperform both CEA and NSE as a biomarker. Moreover, the change in serum CEACAM1 levels was more pronounced in early tumours than in advanced tumours, which may be of great clinical importance. As a result, our data demonstrated that CEACAM1 might be a useful monitor for NSCLC. However, it should be noted that because easy-to-diagnose patients are often enrolled in phase I studies, our results may overestimate accuracy [28]. As we were limited by sample size, future larger prospective studies are needed to validate the prognostic value of serum CEACAM1.

The origin of serum CEACAM1 in NSCLC remains unclear. Previous reports demonstrated that soluble CEACAM1 could be produced by tumour cells and the endothelial cells of angiogenic microvessels [19,38]. The soluble CEACAM1 present in serum comprised membrane-bound isoforms and naturally occurring secreted isoforms. The 3 isoforms of CEACAM1 were found to be in their secreted form, contributing to the serum levels of CEACAM1. Moreover, soluble CEACAM1 was present in serum and has been reported to contain A2 domains [36], corresponding to the membrane-bound isoforms of CEACAM1-4L, CEACAM1-4S and the secreted isoform CEACAM1-4C1. Moreover, it was further demonstrated that apoptosis could induce cleavage of the intracellular and extracellular domains of CEACAM1, resulting in an increased level of soluble CEACAM1 [39]. All of these reports have provided evidence of a number of sources of soluble CEACAM1 in addition to the naturally occurring secreted isoforms [39,40]. Based on the information mentioned above, soluble CEACAM1 may originate from shedding or dead cells in addition to active secretion.

In a continuing study, we further analysed the expression and location of CEACAM1 in NSCLC tissues. By immunohistochemistry, we found strong CEACAM1 staining present in 17 of 21 samples, with CEACAM1 expression localised to the neoplastic epithelium; there was little/no staining in normal tissues. These results significantly supported the up-regulation of CEACAM1 levels in the serum of NSCLC patients. Although 15 of 21 cases showed higher CEACAM1 mRNA levels in tumour tissues than corresponding adjacent tumour-free tissues, no statistically significant difference was found with respect to the mRNA expression level of CEACAM1 in these tissues. The disparity in the CEACAM1 protein and RNA levels in the present study may be due to the differential splicing and proteolytic processing of CEACAM1 after transcription, which needs to be verified in future.

Although there are discrepancies between our findings and previous observations that CEACAM1 was remarkably increased in cancerous tissues of the lung compared with normal lung tissue [31,41], our additional studies of the CEACAM1 S/L isoform expression patterns were in accordance with previous reports. In our research, the CEACAM1-S mRNA expression level and the CEACAM1-S/CEACAM1-L (S: L) ratio were significantly higher in tumour tissues than in normal tissues. The expression of CEACAM1 on microvessels in NSCLC was not found by immunohistochemical staining [24], and human granulocytes, T cells and B cells were reported to only express the CEACAM1-L isoform without CEACAM1-S [42]. CEACAM1-S in the NSCLC tumour tissues appeared to solely derive from tumour cells, whereas CEACAM1-L may not. Thus, the CEACAM1-S RNA levels and CEACAM1-S/L ratios, which are increased in NSCLC, could closely reflect the expression level of tumour cells. Our results indicated that the expression of CEACAM1-S and the CEACAM1-S/CEACAM1-L ratio could be changed without an alteration in the CEACAM1 total expression levels of CEACAM1 in NSCLC. It reinforced the hypothesis that the tumour suppressive or oncogenic effects of CEACAM1 were splice variant-dependent [9,35,43].

## Conclusions

In conclusion, our study strongly suggests the potential use of serum CEACAM1 and the tissue S/L ratio of CEACAM1 as indicators for NSCLC diagnosis. Our research indicated that CEACAM1 may originate from tumour cells. Furthermore, the CEACAM1 isoform expression patterns could change even without changing the quantity of CEACAM1 during oncogenesis. However, the involvement of CEACAM1 in NSCLC and other cancers is complex, and further studies are required. Many studies need to be performed to better understand of the mechanisms that underlie our observations.

## Abbreviations

NSCLC: Non-small-cell lung cancer; CEACAM1: Carcinoembryonic antigen-related cell adhesion molecule 1; CEA: Carcinoembryonic antigen; ITIM: Immunoreceptor tyrosine-based inhibitory motifs; PCR: Polymerase chain reaction; NSE: Neuron-specific enolase; ROC: Receiver operating characteristic; AUC: Area under the curve; RT-PCR: Relative quantitative real time polymerase chain reaction; ELISA: Enzyme-linked immunosorbent assay; HRP: Horseradish peroxidase; TMB: 3,3’,5,5’-tetramethylbenzidine; IOD: Integral optical density.

## Competing interests

The authors declare that they have no competing interests.

## Authors’ contributions

MZ, YD and FG designed the study and wrote the manuscript. YL, YW, CY, YH and WW performed the statistical analyses and experiments. All of the authors contributed to revising the manuscript and approved the final version.

## Pre-publication history

The pre-publication history for this paper can be accessed here:

http://www.biomedcentral.com/1471-2407/13/359/prepub

## Supplementary Material

Additional file 1: Table S1 Clinical and pathological details for the involved patients. Click here for file

Additional file 2: Table S2 Indicators for the diagnostic accuracy of CEACAM1 and tumour markers in lung cancer. Click here for file
