# Supplementary material for: Clinical and experimental studies regarding the expression and diagnostic value of carcinoembryonic antigen-related cell adhesion molecule 1 in non-small-cell lung cancer
Source: BMC Cancer. 2013 Jul 25;13:359. doi: 10.1186/1471-2407-13-359 (PMC3728234; doi:10.1186/1471-2407-13-359)
Supplement: Additional file 1: Table S1 — Clinical and pathological details for the involved patients. [file 1471-2407-13-359-S1.doc]

# Additional file 1: Table S1 Clinical and pathological details for the involved patients

|  | Serum samples  (n=35) | |  | Tissue samples^1^  (n=21) | |  | Tissue samples^2^  (n=13) | |
| --- | --- | --- | --- | --- | --- | --- | --- | --- |
|  | No. | % |  | No. | % |  | No. | % |
| Sex |  |  |  |  |  |  |  |  |
| Male | 16 | 45.7 |  | 12 | 57.1 |  | 7 | 53.8 |
| Female | 19 | 54.3 |  | 9 | 42.9 |  | 6 | 46.2 |
| Stage |  |  |  |  |  |  |  |  |
| IA | 6 | 17.1 |  | 4 | 19.0 |  | 1 | 7.69 |
| IB | 8 | 22.9 |  | 5 | 23.8 |  | 1 | 7.69 |
| IIA | 5 | 14.3 |  | 5 | 23.8 |  | 5 | 38.5 |
| IIB | 0 | 0 |  | 0 | 0 |  | 2 | 15.4 |
| IIIA | 6 | 17.1 |  | 5 | 23.8 |  | 4 | 30.8 |
| IIIB | 1 | 2.85 |  | 0 | 0 |  | 0 | 0 |
| IV | 9 | 25.7 |  | 2 | 9.5 |  | 0 | 0 |
| Grade of differentiation |  |  |  |  |  |  |  |  |
| Well differentiated | 10 | 28.6 |  | 6 | 28.6 |  | 5 | 38.5 |
| Moderately differentiated | 4 | 11.4 |  | 4 | 19.0 |  | 4 | 30.8 |
| Poorly differentiated | 16 | 45.7 |  | 9 | 42.9 |  | 3 | 23.1 |
| Undifferentiated | 5 | 14.3 |  | 2 | 9.5 |  | 1 | 7.69 |

“1” refers to the tissue samples used for the detection of the CEACAM1 mRNA level with quantitative real-time PCR and CEACAM1 protein level with immunohistochemical staining.

“2” refers to tissue samples used to detect the CEACAM1 isoform expression patterns with reverse transcription-PCR.
