# Supplementary material for: Clinical and experimental studies regarding the expression and diagnostic value of carcinoembryonic antigen-related cell adhesion molecule 1 in non-small-cell lung cancer
Source: BMC Cancer. 2013 Jul 25;13:359. doi: 10.1186/1471-2407-13-359 (PMC3728234; doi:10.1186/1471-2407-13-359)
Supplement: Additional file 2: Table S2 — Indicators for the diagnostic accuracy of CEACAM1 and tumour markers in lung cancer. [file 1471-2407-13-359-S2.doc]

# Additional file 2: Table S2 Indicators for the diagnostic accuracy of CEACAM1 and tumour markers in lung cancer

|  | **Lung cancer vs. Normal control** | | | |
| --- | --- | --- | --- | --- |
|  | **Sensitivity (%)** | **Specificity (%)** | **PPV (+)^1^ (%)** | **NPV(-)^2^ (%)** |
| **CEACAM1** | **34/35(97)** | **28/34(82)** | **34/40(70)** | **28/29(95)** |
| **CEA** | **10/35(29)** | **33/34(97)** | **10/11(91)** | **33/58(57)** |
| **NSE** | **7/35(20)** | **33/34(97)** | **7/8(88)** | **33/61(54)** |

“1” PPV (positive predictive value), the proportion of positive test results that are true positives.

“2” NPV (negative predictive value), the proportion of negative test results that are true negatives.
